# The role of automatic defensive responses in the development of posttraumatic stress symptoms in police recruits: protocol of a prospective study

**DOI:** 10.1080/20008198.2017.1412226

**Published:** 2017-12-20

**Authors:** Saskia B. J. Koch, Floris Klumpers, Wei Zhang, Mahur M. Hashemi, Reinoud Kaldewaij, Vanessa A. van Ast, Annika S. Smit, Karin Roelofs

**Affiliations:** ^a^ Donders Institute for Brain, Cognition and Behaviour, Centre for Cognitive Neuroimaging, Radboud University Nijmegen, Nijmegen, The Netherlands; ^b^ Behavioural Science Institute, Radboud University Nijmegen, Nijmegen, The Netherlands; ^c^ Department of Psychology, University of Amsterdam, Amsterdam, The Netherlands; ^d^ Police Academy of the Netherlands, Apeldoorn, The Netherlands

**Keywords:** Freeze–fight–flight responses, trauma, posttraumatic stress disorder, PTSD, neuroimaging, psychophysiology, prospective study, police recruits, aggression, anxiety, Respuestas de congelación-lucha-huida, trauma, trastorno por estrés postraumático, TEPT, neuroimagen, psicofisiología, estudio prospectivo, reclutas de la policía, agresión, ansiedad, 关键词：冻结-战斗-逃跑反应，创伤，创伤后应激障碍，PTSD，神经影像，心理物理，前瞻研究，新募警员，侵犯，焦虑, • The ‘Police-in-Action’ study investigates the role of automatic defensive responses in the development and maintenance of PTSD symptomatology after trauma exposure. • 340 Police recruits are tested before and after trauma exposure during their first emergency aid experiences. • Automatic defensive responses are assessed at both waves at the behavioural, autonomic, endocrine and neural level. • This will enable us to disentangle predisposing from acquired neurobiological abnormalities associated with the development of trauma-related symptoms.

## Abstract

**Background**: Control over automatic tendencies is often compromised in challenging situations when people fall back on automatic defensive reactions, such as *freeze* *–* *fight* *–* *flight* responses. Stress-induced lack of control over automatic defensive responses constitutes a problem endemic to high-risk professions, such as the police. Difficulties controlling automatic defensive responses may not only impair split-second decisions under threat, but also increase the risk for and persistence of posttraumatic stress disorder (PTSD) symptoms. However, the significance of these automatic defensive responses in the development and maintenance of trauma-related symptoms remains unclear due to a shortage of large-scale prospective studies.

**Objective**: The ‘Police-in-Action’ study is conducted to investigate the role of automatic defensive responses in the development and maintenance of PTSD symptomatology after trauma exposure.

**Methods**: In this prospective study, 340 police recruits from the Dutch Police Academy are tested *before* (wave 1; pre-exposure) and *after* (wave 2; post-exposure) their first emergency aid experiences as police officers. The two waves of data assessment are separated by approximately 15 months. To control for unspecific time effects, a well-matched control group of civilians (*n* = 85) is also tested twice, approximately 15 months apart, but without being frequently exposed to potentially traumatic events. Main outcomes are associations between (changes in) behavioural, psychophysiological, endocrine and neural markers of automatic defensive responses and development of trauma-related symptoms after trauma exposure in police recruits.

**Discussion**: This prospective study in a large group of primary responders enables us to distinguish predisposing from acquired neurobiological abnormalities in automatic defensive responses, associated with the development of trauma-related symptoms. Identifying neurobiological correlates of (vulnerability for) trauma-related psychopathology may greatly improve screening for individuals at risk for developing PTSD symptomatology and offer valuable targets for (early preventive) interventions for PTSD.

## Abstract (es)

Planteamiento: El control de las tendencias automáticas a menudo se ve comprometido en situaciones complicadas cuando las personas recurren a las reacciones defensivas automáticas, como las respuestas de ‘congelación-lucha-huida’. La falta de control sobre las respuestas defensivas automáticas inducida por el estrés constituye un problema endémico de las profesiones de alto riesgo, como la policía. Las dificultades para controlar las respuestas defensivas automáticas pueden no solo perjudicar las decisiones inmediatas frente a una amenaza, sino también aumentar el riesgo y la persistencia de los síntomas del trastorno por estrés postraumático (TEPT). Sin embargo, aún no está clara la importancia de estas respuestas defensivas automáticas en el desarrollo y el mantenimiento de los síntomas relacionados con el trauma debido a la escasez de estudios prospectivos a gran escala.

Objetivo: El estudio ‘Policía en acción’ se lleva a cabo para investigar el papel de las respuestas defensivas automáticas en el desarrollo y mantenimiento de la sintomatología del TEPT después de haber sido expuestos a un trauma.

Métodos: en este estudio prospectivo, se pasó un test a 340 reclutas de la policía de la Academia de Policía holandesa antes (onda 1, pre-exposición) y después (onda 2, post-exposición) de sus primeras experiencias de ayuda de emergencia como agentes de policía. Las dos ondas de evaluación de datos están separadas por unos 15 meses. Para controlar los efectos no específicos del tiempo, también se le pasó prueba dos veces a un grupo de control de civiles (*n* = 85), con aproximadamente 15 meses de diferencia, pero sin estar expuesto frecuentemente a eventos potencialmente traumáticos. Los resultados principales son asociaciones entre (cambios en) los marcadores conductuales, psicofisiológicos, endocrinos y neurales de las respuestas defensivas automáticas y el desarrollo de síntomas relacionados con el trauma después de la exposición al trauma en los reclutas de la policía.

Discusión: Este estudio prospectivo en un grupo grande de personal de respuesta a emergencias nos permite distinguir anormalidades neurobiológicas predispuestas y adquiridas en respuestas defensivas automáticas, asociadas con el desarrollo de síntomas relacionados con el trauma. Identificar los correlatos neurobiológicos de (la vulnerabilidad de) la psicopatología relacionada con el trauma puede mejorar en gran medida las pruebas de detección para las personas en riesgo de desarrollar la sintomatología de TEPT y ofrecer objetivos valiosos para intervenciones (preventivas tempranas) para el TEPT. Registrado en el Registro de Holanda: NTR6355

## ABC

背景：在有挑战的情景中,人们通常依赖自动防御反应（比如‘冻结-战斗-逃跑’），而无法有效控制自动反应倾向。由应激引起的对自动防御反应的控制缺乏对高风险职业（例如警察）构成问题。难以控制自动防御反应可能不仅仅损害了威胁情况下的瞬间决策能力，并且同时也增加了创伤后应激障碍（PTSD）的患病风险和症状残留。然而，由于缺乏大规模的回溯研究，这些自动防御反应在创伤相关症状的发展和维持中的重要性还是不清楚的。

目标：‘现役警察’研究旨在考察自动防御反应在创伤暴露后PTSD 症状的发展和保持中的作用。

方法：在这个回溯研究中，340名从荷兰警察学院招募的新警员在他们作为警察第一次应急救援经验之前（第一次，暴露前）和之后（第二次，暴露后）接受测试。 为了控制时间效应的不准确，一个相匹配的市民控制组（N=85）也将在15个月的间隔时间里被测试两次，但他们不会频繁暴露于可能的创伤性事件。主要的结果是行为（改变）、心理物理、内分泌和自动防御反应的神经标记，以及新警员在创伤暴露后的创伤相关症状发展之间的关联。

讨论：这个在大样本的一线救援者中进行的回溯研究使我们可以区分自动防御反应中因素性和习得性的神经生理异常，这些反应和创伤相关症状关联。识别创伤相关心理病理（的易感性）的神经生理关联可能会极大改进对PTSD风险人群的筛查，同时提供对PTSD（早期预防性）干预的宝贵靶点。荷兰试验注册号：NTR6355。

## 1. Background

Control over automatic tendencies is often challenged in threatening situations when people fall back on automatic defensive reactions, such as *freeze* *–* *fight* *–* *flight* responses. Stress-induced lack of control over automatic defensive responses is especially problematic for people in high-risk professions, such as police officers, whose control over automatic responses is essential for optimal performance under threat (Nieuwenhuys, Savelsbergh, & Oudejans, 2012). Moreover, difficulties controlling automatic defensive responses may not only affect split-second decisions under acute threat, but also increase the risk for development and persistence of posttraumatic stress disorder (PTSD) symptoms (Kozlowska, Walker, McLean, & Carrive, 2015). PTSD symptoms are highly prevalent among police officers: it was estimated that over 34% of Dutch police officers experienced PTSD or subsyndromal PTSD symptoms within one year of experiencing a critical incident (Carlier, Lamberts, & Gersons, 1997). Revealing (neurobiological) mechanisms underlying the development and persistence of PTSD symptoms is an important research avenue (Lanius & Olff, 2017), potentially offering modifiable neurobiological targets for (early preventive) interventions for PTSD (Van Zuiden, Kavelaars, Geuze, Olff, & Heijnen, 2013).

**Figure 1.**
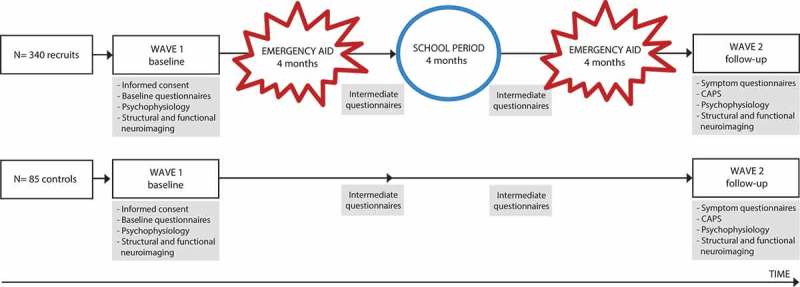
Design of the Police-in-Action study. A total of 340 police recruits and 85 control participants will be tested twice, at baseline (wave 1) and at follow-up (wave 2), which are approximately 15 months apart. Between assessment waves, police recruits (but not controls) experience two emergency aid periods as part of their normal training, each lasting four months. CAPS = clinician-administered PTSD scale.

**Figure 2.**
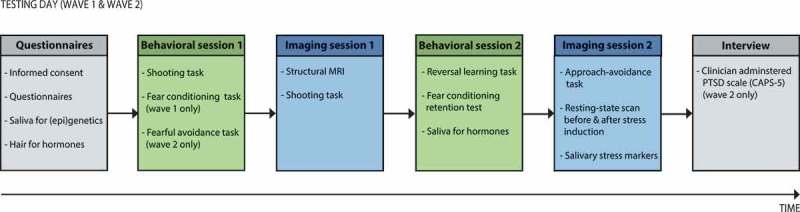
Overview of the testing days. The wave 1 and wave 2 assessments consist of similar testing procedures, except for two minor differences in Behavioural session 1 and the Interview (see figure). MRI = magnetic resonance imaging.

Neurobiological models on the pathophysiology of PTSD describe enhanced activity and connectivity between core nodes of the salience network (SN), such as the amygdala, anterior insula and dorsal anterior cingulate cortex (dACC) (Hayes, Hayes, & Mikedis, 2012; Patel, Spreng, Shin, & Girard, 2012; Pitman et al., 2012). The SN is an intrinsic connectivity network directed at the detection of biologically relevant information in the environment (Seeley et al., 2007), showing increased engagement during the unconscious processing of trauma-related stimuli in PTSD patients, compared to healthy controls (Rabellino et al., 2015). Especially the amygdala is important in fear learning, the detection of threat and orchestrating the expression of fear in coordination with the basal forebrain (Fox, Oler, Tromp, Fudge, & Kalin, 2015). Besides amygdala hyperactivity, PTSD has been associated with hyperactivity in prefrontal structures involved in fear expression, such as the dACC (Hayes et al., 2012; Pitman et al., 2012). Further, brainstem structures, such as the periaqueductal grey (PAG), are involved in physiological responses of automatic defensive mechanisms. For example, PAG activity has been associated with heart rate acceleration (i.e. tachycardia) in response to acute social stress (Wager et al., 2009), as well as with freezing-induced heart rate deceleration (i.e. bradycardia) in response to emotional stimuli (Hermans, Henckens, Roelofs, & Fernández, 2013). In PTSD patients, PAG hyperactivity towards emotional faces with direct vs. averted gaze was observed (Steuwe et al., 2014), and PAG activity towards fearful faces was positively associated with severity of hyperarousal symptoms (Rabellino, Densmore, Frewen, Théberge, & Lanius, 2016). Moreover, enhanced functional connectivity between the PAG and salience processing areas (e.g. dACC and anterior insula) was observed in PTSD (Harricharan et al., 2016; Thome et al., 2017). Neural control over autonomic responses is exerted by various brain regions comprising the central autonomic network, including the amygdala, brainstem, vmPFC and dACC (Critchley, Nagai, Gray, & Mathias, 2011; Etkin, Egner, & Kalisch, 2011; Thayer, Åhs, Fredrikson, Sollers, & Wager, 2012). Autonomic dysregulations in PTSD, as measured with decreased high-frequency heart rate variability (HF-HRV), was associated with altered activity and desynchronized functional connectivity of brain regions involved in autonomic regulation, respectively during presentation of trauma-related stimuli (Rabellino et al., 2017) and during rest (Thome et al., 2017).

Although generally adaptive under threat, amygdala-mediated fear responses should be controlled by the prefrontal cortex in safe, unthreatening contexts. Not surprisingly, the predominant neurocircuitry model of PTSD postulates deficient inhibitory control of the ventromedial prefrontal cortex (vmPFC) over the fear response (Pitman et al., 2012; Rauch, Shin, & Phelps, 2006), characterized with hypo-activity of the vmPFC (Hayes et al., 2012; Patel et al., 2012), as well as diminished functional (Sripada et al., 2012) and structural (Koch et al., 2017) connectivity between the amygdala, vmPFC and hippocampus. Of note, the vmPFC and hippocampus are important nodes of the default mode network (DMN), which is an intrinsic connectivity network engaged during rest and involved in internally focused thought (Greicius, Krasnow, Reiss, & Menon, 2003). During rest, PTSD has been associated with decreased DMN connectivity, and increased functional coupling between nodes of the DMN and SN (Sripada et al., 2012), possibly reflecting symptoms of hypervigilance. During working memory performance, chronic PTSD patients showed both deficient disengagement of the DMN and engagement of the central executive network (CEN), suggesting impaired task-induced switching between major intrinsic connectivity networks (Daniels et al., 2010). Taken together, PTSD has been associated with increased activity in salience processing areas, deficient prefrontal control over the fear response, and aberrant within- and between-network connectivity.

These neurobiological abnormalities presumably result from a combination of predisposing vulnerability factors for the development of PTSD, the effects of trauma and the effects of PTSD (Admon, Milad, & Hendler, 2013). It has been suggested that hyperactivity of salience processing areas (i.e. the amygdala and dACC) is etiologically involved in the development of PTSD, whereas dysfunctions in DMN nodes (i.e. hippocampus and vmPFC) may represent an acquired deficit of PTSD (Admon et al., 2013). Regarding extinction learning, deficient retention of fear extinction was found to be an acquired trait in combat-exposed PTSD patients (Milad et al., 2008). In contrast, increased fear responsiveness (Pole et al., 2009) and deficient fear extinction learning (Lommen, Engelhard, Sijbrandij, Van Den Hout, & Hermans, 2013) as assessed pre-trauma were found to predict development of PTSD symptoms, and may therefore constitute predisposing vulnerability factors for PTSD. Additionally, individual differences in autonomic nervous system (ANS) and hypothalamus–pituitary–adrenal (HPA) axis functioning before and in response to trauma have been related to increased PTSD risk. For example, pre-trauma lower hair cortisol levels (Steudte-Schmiedgen et al., 2015), post-trauma lower basal cortisol levels (Van Zuiden et al., 2013), higher heart rate (Coronas et al., 2011) and lower heart rate variability (Minassian et al., 2015; Shaikh Al Arab et al., 2012) were associated with development of PTSD symptoms. However, results have been inconsistent (Michopoulos, Norrholm, & Jovanovic, 2015; Van Zuiden et al., 2013) and were based on relatively small samples sizes, underlining the need for large prospective studies to disentangle predisposing from acquired neurobiological abnormalities in PTSD (Admon et al., 2013). Furthermore, there is currently limited understanding of the role of automatic defensive responses, such as *freeze* *–* *fight* *–* *flight* responses, in the development and maintenance of PTSD symptoms.

Upon encountering a threatening situation, a parasympathetic state of automatic attentive immobility, characterized by bodily immobility and heart rate deceleration, called the *freeze* response (Fanselow, 1994), facilitates fast risk assessment. During this action preparation stage, the individual is hypervigilant for environmental cues, upon which sympathetically driven *fight-or-flight* responses are selected (Gladwin, Hashemi, Van Ast, & Roelofs, 2016; Löw, Weymar, & Hamm, 2015). Whereas *freezing* involves direct projections from the amygdala to the ventral PAG of the brainstem, the active *fight–flight* responses depend on the dorsal PAG and amygdala projection to the ventral striatum, facilitated by dopaminergic projections (LeDoux, 1998). Freezing is generally considered as adaptive stress-coping mechanism, and the timing of the transition from passive *freeze* to active *fight-or-flight* may be essential for optimal decision-making under threat. Maladaptive, exacerbated freezing was observed in anxious (Roelofs, Hagenaars, & Stins, 2010) and trauma-exposed (Hagenaars, Stins, & Roelofs, 2012) individuals, as well as in adolescents with insecure attachment during infancy (Niermann et al., 2015). Moreover, self-reported tonic immobility during trauma exposure was predictive for the development of PTSD symptoms after sexual assault (Bovin, Jager-Hyman, Gold, Marx, & Sloan, 2008). Of note, however, tonic immobility and freezing are distinct defensive states: freezing prepares an individual for future defensive responses; tonic immobility takes place later in the defensive cascade, when threat is inescapable and no alternative defensive responses remain (Kozlowska et al., 2015; Roelofs, 2017). It has been suggested that aggression is associated with facilitated approach of (social) threat, possibly indicating increased *fight* behaviour, whereas anxiety is related to increased *freeze* and *flight* responses (Niermann, Figner, & Roelofs, 2017). *Flight* reactions can be considered as active avoidance of the threatening cue or situation, which is a cardinal symptom of anxiety disorders and PTSD (American Psychiatric Association, 2013), and – by preventing fear extinction – constitute a major maintaining factor of anxiety (Barlow, 2002; Mowrer, 1960). Taken together, maladaptive automatic defensive responses under threat may account for the development and persistence of posttraumatic symptoms.

Therefore, the main objective of the ‘Police-in-Action’ study is to determine the role of automatic defensive responses in the development of trauma-related symptoms in 340 police recruits, tested *before* (wave 1; pre-exposure) and *after* (wave 2; post-exposure) their first emergency aid experiences as police officers. Automatic defensive responses are assessed at the behavioural, psychophysiological, endocrine and neural level. We aim to identify *pre-existing* vulnerabilities and *acquired* abnormalities in automatic defensive responses related to the development of trauma-related symptomatology. As secondary aim, we aim to gain mechanistic insights into neural and psychophysiological correlates of automatic defensive reactions, based on the baseline assessments in our large group of healthy participants. In line with to the Research Domain Criteria (RDoC) (Insel, 2014), participants will be stratified based on their (trauma-related changes in) automatic defensive responses, and we hypothesize that this stratification will be associated with development of specific trauma-related symptoms, including the DSM-5 PTSD symptom clusters (American Psychiatric Association, 2013) and aggression symptom severity. More specifically, we predict that greater *freeze* and *flight* responses are related to (the development of) anxiety-related symptoms, and that greater *fight* responses are related to (the development of) aggressive symptoms.

## 2. Methods

### 2.1. Study design

The ‘Police-in-Action’ study has a longitudinal design and consists of two waves of data assessment, separated by approximately 15 months. The first assessment wave (wave 1; pre-exposure) takes place *while* police recruits are in the relatively safe environment of training in the police academy. The second assessment wave (wave 2; post-exposure) takes place *after* police recruits have been exposed to aversive events, as part of their first emergency aid services. To assure that observed differences between wave 1 and wave 2 are not due to unspecific time effects, such as test-retest effects, a well-matched control group of civilians is included. These participants are also assessed twice, approximately 15 months apart, but are not expected to be frequently exposed to potentially traumatic events. See Figure 1 for the study design.

### 2.2. Participants

In total, 340 police recruits (25% female) from the Dutch Police Academy and 85 healthy control participants from the Dutch general population are included in this study. Control participants are matched to the police recruits on age, sex and education level. All participants are between 18 and 45 years of age and eligible for MRI. Exclusion criteria include any current psychiatric or neurological disorder, history or current endocrine or neurological treatment, current use of psychotropic medication and current drug or alcohol abuse. Additional exclusion criteria for control participants involve previous experience in law enforcement or an occupation involving potential trauma exposure (e.g. in military or health care). See Table 1 for all inclusion and exclusion criteria. Before study participation, all participants receive oral and written study information and provide written informed consent. The study is conducted in accordance with the principles of the Declaration of Helsinki and has been approved by the Independent Review Board Nijmegen (IRBN), the Netherlands.

**Table 1.** Inclusion and exclusion criteria.

Inclusion criteria
1. Between 18 and 45 years of age
2. Normal or corrected-to-normal vision
3. Normal uncorrected hearing
4. Body mass index between 18.5 and 30
5. Willingness and ability to give written informed consent
**Exclusion criteria**
1. Average use of more than three alcoholic beverages daily
2. Average use of psychotropic medication or recreational drugs weekly or more
3. Use of psychotropic medication
4. Recreational drugs use within 72 hours prior to testing, or alcohol use within 24 hours prior to testing
5. Current psychiatric or neurological disorder, or within the last three months
6. Regular use of systemic corticosteroids
7. Metal objects or fragments in or around the body
8. Medical plaster that cannot be taken off
9. History of neurological treatment or current neurological treatment
10. History of endocrine treatment or current endocrine treatment
11. History of head surgery
12. Current periodontitis
13. Claustrophobia
14. Epilepsy
15. Pregnancy
**Additional exclusion criteria for control participants**
16. Experience in law enforcement or military
17. Training or occupation involving potential trauma exposure, e.g. military or healthcare occupation

### 2.3. Study procedures

#### 2.3.1. Wave 1: baseline visit

During the baseline visit (wave 1), a questionnaire battery is administered, and saliva and hair samples are collected for (epi)genetic and neuroendocrine analyses, respectively. Hereafter, the first behavioural and psychophysiological session consists of a fear conditioning task and shooting task (Gladwin et al., 2016). The fear conditioning task assesses contextual control over cued fear-acquisition, extinction and generalization (Van Ast, Vervliet, & Kindt, 2012), whereas the shooting task assesses shooting decisions under threat that involve a dynamic switch from *freezing* to *fight* behaviour (Gladwin et al., 2016), as well as the switch from danger to safety (Klumpers et al., 2010). In the subsequent functional neuroimaging session, the shooting task is repeated in the MRI scanner. In the second behavioural and psychophysiological session, a probabilistic reversal learning task (Den Ouden et al., 2013) and fear conditioning retention test are administered, to test cognitive flexibility and fear retention from the previous fear-conditioning task, respectively. Finally, during the second fMRI session, (neural) control over emotional actions is investigated with an approach–avoidance task (Roelofs, Minelli, Mars, Van Peer, & Toni, 2009), and resting-state scans are obtained before and after stress induction (Vogel et al., 2015), to assess stress-related alterations in resting-state networks. Saliva samples for endocrinological analyses are collected before the second MRI session, and at several time-points before and after the stress induction to assess stress reactivity.

#### 2.3.2. Intermediate questionnaires

Between the baseline and follow-up measurements, participants are sent online questionnaires every four months assessing the subjective stress experienced in the past months, using the Perceived Stress Scale (PSS) (Cohen, Kamarck, & Mermelstein, 1983) and four visual analogue scales (VAS). For the police recruits, these questionnaires coincide with the termination of their first emergency aid period and subsequent school period. The same questionnaires are sent out to the control participants, with similar timing and frequency.

#### 2.3.3. Wave 2: follow-up

After approximately 15 months, the follow-up visit (wave 2) takes place, consisting of comparable testing procedures as the first testing day. However, some minor differences exist. First, trait questionnaires are not repeated at follow-up, as these constructs are considered to be rather stable. Additionally, during the first behavioural session, the fear conditioning task is adapted to assess only retention of conditioned fear. A fearful avoidance task is added, measuring maladaptive fearful avoidance behaviour coming at the cost of positive outcomes. Finally, a clinical diagnostic interview assessing PTSD symptom severity (clinician-administered PTSD Scale; CAPS-5) (Boeschoten et al., 2014) is administered within one week after the second testing day. See Figure 2 for an overview of the testing days.

### 2.4. Primary outcome measures

The primary outcomes of this study are associations between (changes in) biomarkers of automatic defensive reactions and changes in trauma-related symptomatology after emergency aid experience in police recruits.

#### 2.4.1. Trauma-related symptomatology

Severity of PTSD symptoms is measured with the PTSD Checklist for DSM-5 (PCL-5) (Boeschoten, Bakker, Jongedijk, & Olff, 2014; Weathers et al., 2013). Participants self-rate the severity of all DSM-5 PTSD symptoms in the last month, ranging from 0 (absent) to 4 (extreme/incapacitating). The revised Life Events Checklist for DSM-5 (LEC-5) is administered in combination with the PCL-5 to screen for potentially traumatic events. Severity of aggressive symptoms in the past six months is assessed with the 30-item impulsive/premeditated aggression scale (IPAS) (Stanford, Houston, Villemarette-Pittman, & Greve, 2003). Additionally, reactive and proactive aggressive symptoms in the past six months are assessed with the reactive proactive aggression questionnaire (RPQ) (Raine et al., 2006). These questionnaires are administered at wave 1 and wave 2.

#### 2.4.2. Automatic defensive responses

*Shooting task*. To assess psychophysiological and neural correlates of decisions under threat, a shooting task is employed (Gladwin et al., 2016). During this task, one of two opponents is presented on screen, signalling impending shooting decisions either under threat of shock or safety. After an anticipation interval, the opponent either draws a gun aimed at the participant (requiring fast reactions) or a phone (requiring action inhibition). There is a safe and a threatening opponent, the latter predicting threat of shock in case of incorrect responses. During the behavioural session, the task is performed on a stabilometric force platform to assess body sway, while heart rate is simultaneously measured (Gladwin et al., 2016). A similar shooting task is administered in the MRI scanner. We hypothesize that maladaptive defensive reactions – including the dynamics of the initial parasympathetic freezing response to the sympathetic act of shooting and the successful control over prepotent shooting responses – are related to (the development of) PTSD symptomatology.

*Fear conditioning task*. To study contextual control over automatic fear and its generalization (Maren, Phan, & Liberzon, 2013), we employ a fear conditioning paradigm in which the background contexts can disambiguate the meaning of a threat cue (Van Ast et al., 2012). Participants are instructed to predict the occurrence of electrical stimulation based on the combination of cue and context. To create danger and safe contexts, an image of a man (cue) is followed by electrical stimulation when presented in an image of an office (threat context), while the same cue is never paired with electrical stimulation in another office (safe context). Immediately following this acquisition phase, a generalization test phase takes place, during which presentations of the cue in the safe and threat contexts are alternated with presentations of the cue in a new – ambiguous – office context (generalization context). After approximately three hours, retention of contextual modulation of the cued fear memories is tested. We expect that enhanced fear generalization across contexts pre-trauma predicts the development of PTSD symptoms after the emergency aid.

*Probabilistic reversal learning task*. To assess the ability to flexibly adapt behaviour in a changing environment, participants perform a probabilistic reversal learning task (Den Ouden et al., 2013). During each trial, participants select one of two presented stimuli (stimulus A and B), which are probabilistically associated with reward and punishment (i.e. positive and negative emoticons). During the first half of the task, stimulus A is rewarded in 70% of trials and punished in 30% of trials (and vice versa for stimulus B). During the second half of the task, the reinforcement contingencies are reversed. Participants should select the stimulus most consistently linked to a rewarding outcome. We hypothesize that diminished cognitive flexibility to adapt behaviour is associated with (the development of) PTSD symptoms.

*Fearful avoidance task*. The avoidance of fearful situations is tested in a paradigm that allows subjects to avoid threatening situations with relatively smaller or bigger costs. In the fearful avoidance task, subjects can either approach or avoid situations with a potential threat of aversive electrical stimulation, but also a potential monetary reward. These situations are varied in a 2 (threat, safe context) x 2 (low, high reward) design, leading to fluctuating degrees of approach–avoid conflict. Subjects are informed that upon approach there is a 50% chance of either outcome. Outcome parameters are the choice behaviour, as well as fear-potentiated startle responses prior to the decision, assessing fear responses. We expect that greater maladaptive avoidance behaviour, especially during safety and at the cost of high potential reward, is associated with PTSD symptoms after trauma exposure.

*Approach–avoidance task*. To test whether (development of) PTSD symptoms is associated with reduced (neural) control over automatic action tendencies to approach positive (happy) and avoid negative (angry) emotional faces, we administer the approach–avoidance task (AAT) during functional MRI scanning (Roelofs et al., 2009). During affect-congruent trials, participants should approach happy faces and avoid angry faces, by pulling a joystick towards themselves or pushing a joystick away, respectively. During the affect-incongruent trials, participants should override their automatic tendencies by approaching angry and avoiding happy faces. We hypothesize that deficient prefrontal control over automatic action tendencies during incongruent vs. congruent responses is associated with the (development of) PTSD symptoms after trauma.

*Resting-state before and after stress induction*. To assess stress-induced changes in autonomic and HPA axis responding, and associated alterations in functional connectivity networks, resting-state scans are acquired before and after stress induction using the Socially Evaluated Cold Pressor Task (SECPT) and mental arithmetic (MA) task (Schwabe, Haddad, & Schachinger, 2008; Vogel et al., 2015). During the stress induction, participants immerse their right foot in ice water (0–3°C) for as long as possible, with a maximum of three minutes. Participants look into a camera while being watched by two experimenters (one wearing a white laboratory coat), acting neutral and non-supportive. Hereafter, the stress induction continues with a short, but difficult, MA task, in which participants count aloud backwards from 2053 in 17-step sequences and have to start anew whenever they make a mistake. This combination of physical stress (i.e. foot in ice water) along with social evaluation and mental arithmetic has previously produced reliable increases in negative mood and HPA axis reactivity (Schwabe et al., 2008; Vogel et al., 2015). We hypothesize that maladaptive (i.e. blunted or exaggerated) neural, endocrine and autonomic stress responses are associated with (the development of) PTSD symptoms after trauma exposure in police recruits.

### 2.5. Secondary outcome measures

#### 2.5.1. (Epi)Genetics

To be able to assess the effects of genetic variation on our main outcome measures, and potential environmental influences on gene expression, we collect saliva using Oragene DNA collection kits (DNA Genotek Inc., Ottawa, Canada) and buccal epithelial cells using Catch-All sample collection swabs (Epicentre, Madison, USA), allowing for genetic and epigenetic measurements. Additionally, we collect saliva using Oragene RNA collection kits (DNA Genotek Inc., Ottawa, Canada), enabling assessment of long non-coding RNA, mRNA and microRNA levels of salivary-expressed genes.

#### 2.5.2. Endocrine markers

To assess baseline salivary cortisol and testosterone levels, participants fill one Salicap (IBL, Hamburg, Germany) with saliva during the first and second testing days. Furthermore, Salivettes (Sarstedt, Rommelsdorf, Germany) are collected at various time-points before (two samples) and after (three samples) stress induction with the SECPT and MA task, to assess stress-related changes in cortisol and alpha-amylase concentrations. To obtain long-term endocrine correlates of PTSD symptoms and traumatization, levels of cortisol and other steroid hormones are assessed in hair, both at wave 1 and wave 2 (Stalder & Kirschbaum, 2012; Steudte-Schmiedgen, Kirschbaum, Alexander, & Stalder, 2016). We collect 3 cm hair strand from the lower part of the back of the head, reflecting cumulative secretion over the previous three-month period.

#### 2.5.3. Questionnaires

We administer several questionnaires assessing mental health outcomes (e.g. anger, anxiety and depressive symptoms), (childhood) trauma history and other individual characteristics which may influence our main outcomes of interests (e.g. attachment security and social support) (see Supplemental data for a complete overview of all questionnaires). Additionally, demographic characteristics such as age, education level, ethnic background, medical history, smoking, alcohol and drug use are collected.

#### 2.5.4. Assessments at the police academy

During the intake procedure at the Dutch Police Academy, several assessments including the Dutch NEO Five-Factor Inventory personality questionnaire (NEO-FFI-NL) (Hoekstra, Fruyt, & Ormel, 2007) and cognitive and physical ability tests are conducted to screen applicants for eligibility to become a police officer. After admission to the police academy, physical fitness of each recruit is additionally tested as part of a physical training course, assessing both muscular strength and aerobic endurance.

### 2.6. Statistical analyses: primary outcome measures

To identify predisposing vulnerability factors for the development of PTSD symptoms, we will test the predictive value of baseline behavioural, psychophysiological, endocrine and neural automatic defensive responses (wave 1) for development of PTSD symptoms after trauma exposure (Δ PTSD symptoms wave 2 – wave 1), using multiple regression analyses. Additionally, we will test for acquired changes in behavioural, psychophysiological, endocrine and neural automatic defensive responses (Δ biomarkers wave 2 – wave 1) as a consequence of trauma exposure and/or the development of PTSD and aggression symptoms (Δ PTSD symptoms wave 2 – wave 1) with multiple regression analyses. *p*-values < .05 (two-sided) will be considered statistically significant.

## 3. Discussion

This study aims to investigate the role of automatic defensive reactions in the development and maintenance of trauma-related psychopathology. Given the high incidence of (subsyndromal) PTSD symptoms in police officers (Carlier & Gersons, 1992) and insufficient response to currently available psychotherapies for PTSD (Bradley, Greene, Russ, Dutra, & Westen, 2005), revealing neurobiological mechanisms underlying the development and persistence of trauma-related psychopathology is highly relevant. The approach of our study has several noteworthy characteristics. First, we will focus on the role of automatic defensive reactions in the development of trauma-related psychopathology. Although automatic defensive responses, such as *freeze* *–* *fight* *–* *flight* tendencies, are suggested to form a major maintaining factor of PTSD (Kozlowska et al., 2015), the role these responses in the development and persistence of PTSD symptoms remains largely unknown. Another characteristic of our study is that we adopt a transdiagnostic approach, investigating intermediate phenotypes of posttraumatic anxiety and aggressive symptoms, rather than relying on a full-blown PTSD diagnosis according to distinct diagnostic categories. Moreover, our longitudinal study allows for disentangling predisposing from acquired neurobiological abnormalities in the development and persistence of PTSD, which may break ground for early symptom detection and preventive interventions, by improving screening for recently trauma-exposed individuals at increased PTSD risk (Michopoulos et al., 2015). As the vast majority of trauma-exposed individuals does not develop PTSD, the current notion is that only individuals at increased risk of PTSD should be targeted with preventive interventions (Kearns, Ressler, Zatzick, & Rothbaum, 2012). Moreover, mechanistic insights into neurobiological correlates of (development of) PTSD could guide development of novel or improved treatment strategies for PTSD.

As the majority of our participants will only develop subclinical PTSD symptoms, the generalizability of our results to the development of full-blown PTSD may be limited. The incidence of current duty-related PTSD in police officers has been found to be 7–19% (Gersons, 1989): among 262 Dutch police officers interviewed at two weeks, three months, and 12 months after experiencing a critical incident, 34% suffered from PTSD or subsyndromal PTSD symptoms at some point during the study (Carlier et al., 1997). Moreover, over 30% of young, inexperienced police recruits developed stress-related symptoms after being exposed to a life-threatening situation (Maguen et al., 2009). Importantly, even with the majority of our participants only developing subclinical PTSD symptoms, we will be able to assess how fundamental biological systems related to automatic defensive responses may underlie individual differences in the development of trauma-related psychopathology. Using a transdiagnostic approach, stratification of participants based on biomarkers of automatic defensive reactions will be used to predict the development of (subclinical) trauma-related symptoms.

Taken together, this prospective study will enable us to distinguish predisposing from acquired abnormalities in automatic defensive responses at the behavioural, psychophysiological, endocrine and neural level associated with the development of trauma-related symptoms in a large group of primary responders. Identifying neurobiological correlates of (risk for) PTSD symptomatology may improve screening for individuals at risk for developing PTSD symptoms, and offer valuable targets for (early preventive) interventions of PTSD.

## Supplementary Material

Supplementary material
