# Supplementary material for: The role of automatic defensive responses in the development of posttraumatic stress symptoms in police recruits: protocol of a prospective study
Source: Eur J Psychotraumatol. 2017 Dec 20;8(1):1412226. doi: 10.1080/20008198.2017.1412226 (PMC5757225; doi:10.1080/20008198.2017.1412226)
Supplement: Supplementary material [file ZEPT_A_1412226_SM3399.docx]

**Supplementary Material**

**The role of automatic defensive responses in the development of posttraumatic stress symptoms in police recruits: protocol of a prospective study**

**Complete overview of questionnaires**

We administer several questionnaires assessing mental health outcomes, (childhood) trauma history, and other individual characteristics, which may influence our main outcomes of interests (see below). Additionally, demographic characteristics such as age, education level, ethnic background, medical history, smoking, alcohol and drug use are collected.

***Mental health outcomes – self-reported measures***

Trait and state anger are assessed using the State-Trait Anger Expression Inventory (Spielberger, 1988), consisting of two 10-item subscales. The Trait-Anger Scale (STAXI-trait) assesses an individual’s disposition to experience anger in general, from 1 (almost never) to 4 (almost always). The State-Anger Scale (STAXI-state) assesses an individual’s feelings of anger at the current moment, from 1 (not at all) to 4 (very much). Both scales have been shown to have good psychometric properties (Spielberger, 1988). Both questionnaires are administered at wave 1, and the STAXI-state is repeated at wave 2.

State and trait anxiety are measured with the Spielberger State-Trait Anxiety Inventory (Spielberger, Gorsuch, & Lushene, 1970; Spielberger, 1983), consisting of two 20-item self-report scales assessing an individual’s general anxiety proneness (STAI-trait) and current levels of anxiety (STAI-state). Items are rated on a Likert scale ranging from 1 (almost never/ not at all) to 4 (almost always/ very much) and summed for a total score (range: 20-80). The Dutch version has been validated (van der Ploeg, 1980). Both questionnaires are administered at wave 1, and the STAI-state is repeated at wave 2.

The Beck Depression Inventory (BDI) (Beck, Ward, Mendelson, Mock, & Erbaugh, 1961) is administered at wave 1 and wave 2 to assess severity of depressive symptoms in the past week. The BDI is a well-established 21-item (4-point scale: 0-3) questionnaire to assess self-reported depressive symptoms, with good reliability and validity (Beck, Steer, & Carbin, 1988).

To assess (changes in) current mood, the Dutch version of the Positive and Negative Affect Scale (PANAS) (Watson, Clark, & Tellegen, 1988) is administered at wave 1 and 2. The PANAS is a 20-item self-report measure consisting of two mood scales: positive affect and negative affect. Items are rated on a 5-point scale (1 = very slightly or not at all; 5 = extremely). The PANAS has good psychometric properties (Crawford & Henry, 2004) and is administered to assess current mood at the start of each testing day, and to assess stress-induced mood changes after the Socially Evaluated Cold Pressure Task (SECPT) and mental arithmetic (MA) task.

Visual analogue scales (VAS) are used to assess feelings of irritability, happiness, stress and anxiety in the past two months on a 9-point scale ranging from 1 (not at all) to 9 (extremely). Additionally, perceived stress in the past two months is assessed with the Perceived stress scale (PSS) (Cohen, Kamarck, & Mermelstein, 1983), consisting of 14-items ranging from 0 (never) to 4 (very often). The PSS has adequate reliability and is associated with various mental health outcomes (Cohen et al., 1983). Both the VAS and PSS are administered at wave 1 and wave 2, and are part of the intermediate online questionnaires assessing perceived stress during the emergency aid periods.

***Mental health outcomes – clinician-rated***

At follow-up, we assess PTSD symptom severity with an established clinical diagnostic interview: the clinical administrated PTSD Scale (CAPS-5) (Blake et al., 1995; Weathers et al., 2013; Dutch translation: Boeschoten et al., 2014). PTSD severity is assessed using the four DSM-5 PTSD symptom clusters: intrusions (5 symptoms), avoidance (2 symptoms), negative alterations in cognition and mood (7 symptoms) and alterations in arousal and reactivity (6 symptoms). Severity is rated for each symptom, ranging from 0 (absent) to 4 (extreme/ incapacitating). The CAPS is administered by telephone at a convenient time for the participant, within one week after the second testing day. Administering the CAPS by telephone has shown high agreement with face-to-face interviews (Aziz & Kenford, 2004).

***Trauma history & emergency aid experience***

To assess police work-related trauma incidence before and during the emergency aids, participants complete the Police Life Events Scale (PLES) (Carlier & Gersons, 1992). The PLES is a list of 40 possible traumatic police work-related events and can be divided into two categories: extremely violent (e.g., shootings or escalating riot situations) and extremely depressing (e.g., finding a corpse or being confronted with severely mutilated victims). The reliability coefficient for the total score is good (Carlier & Gersons, 1992). The PLES is administered at wave 1 and wave 2.

History of childhood trauma is assessed at wave 1 with the Dutch version of the Childhood Trauma Questionnaire-Short Form (CTQ-SF) (Bernstein et al., 2003). This is a 25-item retrospective self-report questionnaire assessing childhood maltreatment on five dimensions: (1) Physical Abuse, (2) Emotional Abuse, (3) Sexual Abuse, (4) Physical Neglect, and (5) Emotional Neglect. Items are scored from 1 (never true) to 5 (very often true). The CTQ-SF has good criterion-related validity (Bernstein et al., 2003) and the Dutch translation has been validated (Thombs, Bernstein, Lobbestael, & Arntz, 2009).

Furthermore, we assess the occurrence of 21 potentially threatening life events with the List of Threatening Experiences (LTE) (Brugha & Cragg, 1990). For each event, participants are asked to indicate whether they have experienced this event (1) before the age of 16, (2) after the age of 16 until one year ago, (3) one year ago or (4) never. The LTE has high test-retest reliability and validity (Brugha & Cragg, 1990), and is administered at wave 1 and 2.

***Other participant characteristics***

The attentional control scale (ACS) (Derryberry & Reed, 2002) is a 20-item self-report questionnaire measuring attentional focusing (9 items) and attentional shifting/disengagement (11 items), on a 4-point scale from 1 (almost never) to 4 (always). Higher scores indicate better attentional control. The ACS has good convergent validity (Derryberry & Reed, 2002) and is administered at wave 1.

Trait impulsivity is assessed at wave 1 with the Barratt Impulsiveness Scale (BIS-11) (Patton, Stanford, & Barratt, 1995), which is a 30-item self-report questionnaire, on a 4-point scale from 1 (rarely/never) to 4 (almost always/always). The BIS-11 has good test-retest reliability and good internal consistency (Stanford et al., 2009).

The Social Support Inventory (SSI) (Timmerman, Emanuels-Zuurveen, & Emmelkamp, 2000) is a 20-item questionnaire assessing the perceived adequacy of social support on a 5-point scale ranging from 1 (much too little support) to 5 (much too much support). The SSI has good psychometric properties (Timmerman et al., 2000) and is administered at wave 1 and wave 2, allowing us to measure possible changes in perceived social support.

Attachment style is measured using the Dutch translation of the Experiences in Close Relationships Scale-revised (ECR-R) (Brennan, Clark, & Shaver, 1998). This questionnaire consists of two 18-items subscales: Attachment Anxiety (i.e. anxiety about abandonment) and Attachment Avoidance (i.e. avoidance of closeness). Items are scored on a 7-point likert scale ranging from 1 (completely disagree) to 7 (completely agree). The ECR-R has high reliability and validity (Ravitz, Maunder, Hunter, Sthankiya, & Lancee, 2010) and is administered at wave 2.

To assess (changes in) alcohol use in the past year, the Alcohol Use Disorders Identification Test (AUDIT) (Saunders, Aasland, Babor, de la Fuente, & Grant, 1993) is administered at wave 1 and wave 2. The AUDIT consists of 10 items, on a 4-point scale. Total AUDIT scores can categorize individuals into low-risk drinking or abstinence, medium-risk drinking, high-risk drinking, and risk for alcohol dependence. The AUDIT has good psychometric properties, such as high internal consistency (Allen, Litten, Fertig, & Babor, 1997).

**References**

Allen, J. P., Litten, R. Z., Fertig, J. B., & Babor, T. (1997). A review of research on the Alcohol Use Disorders Identification Test (AUDIT). *Alcoholism, Clinical and Experimental Research*, *21*(4), 613–9. Retrieved from http://www.ncbi.nlm.nih.gov/pubmed/9194913

Aziz, M. a, & Kenford, S. (2004). Comparability of telephone and face-to-face interviews in assessing patients with posttraumatic stress disorder. *Journal of Psychiatric Practice*, *10*(5), 307–13. http://doi.org/10.1097/00131746-200409000-00004

Beck, A. T., Steer, R. A., & Carbin, M. G. (1988). Psychometric properties of the Beck Depression Inventory: Twenty-five years of evaluation. *Clinical Psychology Review*, *8*(1), 77–100. http://doi.org/10.1016/0272-7358(88)90050-5

Beck, A. T., Ward, C. H., Mendelson, M., Mock, J., & Erbaugh, J. (1961). An inventory for measuring depression. *Archives of General Psychiatry*, *4*, 561–571.

Bernstein, D. P., Stein, J. A., Newcomb, M. D., Walker, E., Pogge, D., Ahluvalia, T., … Zule, W. (2003). Development and validation of a brief screening version of the Childhood Trauma Questionnaire. *Child Abuse & Neglect*, *27*(2), 169–90. Retrieved from http://www.ncbi.nlm.nih.gov/pubmed/12615092

Blake, D. D., Weathers, F. W., Nagy, L. M., Kaloupek, D. G., Gusman, F. D., Charney, D. S., & Keane, T. M. (1995). The development of a Clinician-Administered PTSD Scale. *Journal of Traumatic Stress*, *8*(1), 75–90. Retrieved from http://www.ncbi.nlm.nih.gov/pubmed/7712061

Boeschoten, M. A., Bakker, A., Jongedijk, R. A., Elzinga, B. M., Rademaker, A. R., & Olff, M. (2014). *Clinician Administered PTSD Scale for DSM-5 – Dutch version*. Arq Psychotrauma Expert Groep, Diemen.

Brennan, K. A., Clark, C. L., & Shaver, P. R. (1998). Self-report measurement of adult romantic attachment: An integrative overview. In J. A. Simpson & W. S. Rholes (Eds.), *Attachment theory and close relationships* (pp. 46–76). New York: Guilford press.

Brugha, T. S., & Cragg, D. (1990). The List of Threatening Experiences: the reliability and validity of a brief life events questionnaire. *Acta Psychiatrica Scandinavica*, *82*(1), 77–81. Retrieved from http://www.ncbi.nlm.nih.gov/pubmed/2399824

Carlier, I., & Gersons, B. (1992). Development of a scale for traumatic incidents in police work. *Psychiatrica Fennica.*, *23*, 59–70.

Cohen, S., Kamarck, T., & Mermelstein, R. (1983). A Global Measure of Perceived Stress. *Journal of Health and Social Behavior*, *24*(4), 385. http://doi.org/10.2307/2136404

Crawford, J. R., & Henry, J. D. (2004). The Positive and Negative Affect Schedule (PANAS): Construct validity, measurement properties and normative data in a large non-clinical sample. *British Journal of Clinical Psychology*, *43*(3), 245–265. http://doi.org/10.1348/0144665031752934

Derryberry, D., & Reed, M. A. (2002). Anxiety-related attentional biases and their regulation by attentional control. *Journal of Abnormal Psychology*, *111*(2), 225–36. Retrieved from http://www.ncbi.nlm.nih.gov/pubmed/12003445

Patton, J. H., Stanford, M. S., & Barratt, E. S. (1995). Factor structure of the Barratt impulsiveness scale. *Journal of Clinical Psychology*, *51*(6), 768–74. Retrieved from http://www.ncbi.nlm.nih.gov/pubmed/8778124

Ravitz, P., Maunder, R., Hunter, J., Sthankiya, B., & Lancee, W. (2010). Adult attachment measures: A 25-year review. *Journal of Psychosomatic Research*, *69*(4), 419–432. http://doi.org/10.1016/j.jpsychores.2009.08.006

Saunders, J. B., Aasland, O. G., Babor, T. F., de la Fuente, J. R., & Grant, M. (1993). Development of the Alcohol Use Disorders Identification Test (AUDIT): WHO Collaborative Project on Early Detection of Persons with Harmful Alcohol Consumption--II. *Addiction (Abingdon, England)*, *88*(6), 791–804. Retrieved from http://www.ncbi.nlm.nih.gov/pubmed/8329970

Spielberger, C. (1983). *Manual for the State-Trait Anxiety Inventory (form Y)*. Palo Alto, California: Consulting Psychologists Press.

Spielberger, C. (1988). *Manual for the State-Trait Anger Expression Inventory (STAXI)*. Odessa, Florida: Psychological Assessment Resources.

Spielberger, C., Gorsuch, R., & Lushene, R. (1970). *Manual for the state-trait anxiety inventory*. Palo Alto, California: Consulting Psychologist Press. Retrieved from https://ubir.buffalo.edu/xmlui/handle/10477/2895

Stanford, M. S., Mathias, C. W., Dougherty, D. M., Lake, S. L., Anderson, N. E., & Patton, J. H. (2009). Fifty years of the Barratt Impulsiveness Scale: An update and review. *Personality and Individual Differences*, *47*(5), 385–395. http://doi.org/10.1016/j.paid.2009.04.008

Thombs, B. D., Bernstein, D. P., Lobbestael, J., & Arntz, A. (2009). A validation study of the Dutch Childhood Trauma Questionnaire-Short Form: Factor structure, reliability, and known-groups validity. *Child Abuse & Neglect*, *33*(8), 518–523. http://doi.org/10.1016/j.chiabu.2009.03.001

Timmerman, I. G. H., Emanuels-Zuurveen, E. S., & Emmelkamp, P. M. G. (2000). The social support inventory (SSI): a brief scale to assess perceived adequacy of social support. *Clinical Psychology & Psychotherapy*, *7*(5), 401–410. http://doi.org/10.1002/1099-0879(200011)7:5<401::AID-CPP253>3.0.CO;2-I

Van der Ploeg, M. H. (1980). Validity of the Zelf-Beoordelings-Vragenlijst (A Dutch version of the Spielberger State-Trait Anxiety Inventory). *Nederlands Tijdschrift Voor de Psychologie En Haar Grensgebieden*. Retrieved from http://psycnet.apa.org/psycinfo/1981-22544-001

Watson, D., Clark, L. A., & Tellegen, A. (1988). Development and validation of brief measures of positive and negative affect: the PANAS scales. *Journal of Personality and Social Psychology*, *54*(6), 1063–70. Retrieved from http://www.ncbi.nlm.nih.gov/pubmed/3397865

Weathers, F. W., Blake, D. D., Schnurr, P. P., Kaloupek, D. G., Marx, B. P., & Keane, T. M. (2013). The Clinician-Administered PTSD Scale for DSM-5 (CAPS-5). Interview available from the National Center for PTSD at www.ptsd.va.gov.
